# Dietary inflammatory index and risk of non-alcoholic fatty liver disease and advanced hepatic fibrosis in US adults

**DOI:** 10.3389/fnut.2023.1102660

**Published:** 2023-01-25

**Authors:** Zhongmian Zhang, Lan Wang, Zili Lin, Weitian Yan, Jiaqin Chen, Xiyan Zhang, Wangyu Ye, Jian Li, Zhihong Li

**Affiliations:** ^1^Dongzhimen Hospital, Beijing University of Chinese Medicine, Beijing, China; ^2^The First Affiliated Hospital of Yunnan University of Chinese Medicine, Kunming, Yunnan, China; ^3^Department of Histology and Embryology, Beijing University of Chinese Medicine, Beijing, China

**Keywords:** dietary inflammatory index, non-alcoholic fatty liver disease, advanced hepatic fibrosis, NHANES, population-based study

## Abstract

**Background and aims:**

This study aims to investigate whether the Dietary Inflammatory Index (DII) is associated with non-alcoholic fatty liver disease (NAFLD) and advanced hepatic fibrosis (AHF) among non-institutionalized adults in the United States.

**Methods:**

Utilizing data from the National Health and Nutrition Examination Survey (NHANES) from 2005 to 2016, a total of 10,052 adults aged ≥18 years were included in the analysis. We used multivariable analysis, controlling for demographic variables, to evaluate the association between DII and NAFLD and AHF, a restricted cubic spline (RCS) was used to model the non-linear relationship between DII and NAFLD.

**Results:**

For 10,052 participants, DII ranges from -4.63 to 5.47. Compared with quartile 1, higher DII group were associated with higher levels of female, separated/divorced, lower education level, heavy alcohol use, current smoke status, BMI, poverty income ratio, and waist circumference. DII also showed a significantly positive correlation with ALT, AST. In the fully adjusted multivariable model, DII was positively associated with the presence of NAFLD (OR 1.09, 1.06–1.13 CI, *p* trend <0.0001), and AHF (OR 1.15, 1.07–1.23 CI, *p* trend <0.001). The association remained statistically significant after stratified by gender in terms of NAFLD, but in case of AHF only in males (Q4 vs. Q1: OR 2.68, 1.63–4.41 CI, *p* trend <0.0001) was statistically significant. In the RCS models, the relation of DII and NAFLD started increase rapidly until around 1.80 and then started relatively flat afterward.

**Conclusion:**

Higher pro-inflammatory level was associated with higher risk of NAFLD in males and females, and with higher risk of AHF in males but not in females. Therefore, strategies to promote an Zhang anti-inflammatory diet should be considered to prevent and ameliorate NAFLD and AHF in adults.

## Introduction

Non-alcoholic fatty liver disease (NAFLD) has become the most common chronic liver disease among adults in the United States, with a prevalence of 30–40% (1). The spectrum of NAFLD is ranging from variable degrees of simple steatosis to non-alcoholic steatohepatitis (NASH) (2) with varying amounts of fibrosis and cirrhosis (3, 4), And at least one third of patients with NAFLD will progress to NASH (5) which is characterized by hepatic fat accumulation coincidental with inflammation and potential of advanced hepatic fibrosis (AHF) (6). In patients with NAFLD and AHF (7), liver-related mortality is higher. It has been shown that NAFLD (8) and AHF are associated with hepatic and systemic inflammation.

Dietary modification is a significant impact on liver health (9) and is one of the main modulators of sub-clinical inflammation (10). The overall effect of diet on inflammatory potential can be quantified by Dietary Inflammatory Index (DII), a literature-derived dietary index that was developed to predict inflammation. DII can be used by any population that has dietary data, as it is standardized to global dietary intakes (11). Thus far, a few studies have revealed a correlation between DII and obesity (12), metabolic syndrome (13), cardiovascular diseases (14) and all-cause mortality (15). Moreover, it has been shown that there was an association between DII and hepatic health, Cantero et al. (16) showed putative anti-inflammatory components could specifically ameliorate NAFLD manifestations. However, there were also inconsistent conclusion, Ramírez-Vélez et al. (17) found that the transient elastography parameters, including liver stiffness measure (LSM) and controlled attenuation parameter (CAP), which are markers of hepatic fibrosis and steatosis, respectively, were not correlated with the anti-inflammatory diet profile. To data, there is no epidemiological evidence available about the association between DII and NAFLD or AHF.

Therefore, we aim to assess the cross-sectional relationships between DII and risk of NAFLD and AHF, and the difference between males and females using data from the National Health and Nutrition and Examination Surveys (NHANES). We hypothesized that increased consumption of a pro-inflammatory diet would associate higher NAFLD or AHF risk.

## Materials and methods

### Study cohort

The NHANES is a complex, stratified, 4-stage survey design, and probability-cluster designed program conducted by the National Center for Health Statistics (NCHS) (18), which aimed to evaluate the health and nutritional status of adults and children in the US. The National Center for Health Statistics Institutional Review Board and Ethics Review Board has continuously approved the NHANES study since 1999. Using deidentified data to conduct the secondary analysis was officially classified as exempt by the Albert Einstein College of Medicine Institutional Review Board. There is no need to provide specific written consent for this secondary analysis of existing data. We drafted this report in accordance with the Strengthening the Reporting of Observational Studies in Epidemiology (STROBE) reporting guideline for cross-sectional studies (19).

Data from six cycles of the NHANES that extracted during 2005–2016 were included in this analysis. All participants aged ≥18 years who completed the full 24 h dietary history were included in the cohort. Participants were excluded if they: (1) had unreliable dietary recall status; (2) missed information included fatty liver index (FLI) and NAFLD fibrosis score (NFS), could not define NAFLDa nd AHF; (3) had elevated alcohol intake (>21 standard drinks per week in males; or >14 standard drinks per week in females) (20); (4) had self-reported cancer; (5) were pregnant women; (6) had positive hepatitis B surface antigen, or hepatitis C virus RNA (21).

### Primary exposure

Dietary inflammatory index is a potential tool to evaluate the anti-inflammatory or pro-inflammatory tendency of an individual’s diet (22), which development and validation has been presented in detail elsewhere (11, 23). To eliminate the effects of different total energy intake, we used energy-standardized version of world database to adjust the intake of each food parameter by 1,000 kcal. The participants’ DII score was related to Z-score which was created to express an individual’s exposure relative to the “standard global mean.” Z-score is equal to the value of adjusted participant’s intake subtract the adjusted global daily mean intake, divided by its standard deviation, then converted Z-score to a percentile score and multiplied by two and subtracted from one, next multiplied the percentile score by the score of inflammatory effect of corresponding food parameter. Finally, all the specific DII scores of a food parameter are summed to create the overall DII score for an individual (11). Higher DII scores indicate more pro-inflammatory diets, and lower DII scores indicate more anti-inflammatory diets. We analyzed DII score as a continuous variable, dividing the total sample into quartiles to analysis.

### Outcome definitions

Fatty liver index and NFS have been used for non-invasive diagnostic indexes for liver disease detection. FLI, a validated diagnostic index, was used to define NAFLD (24). FLI score ≥60 was assumed to have NAFLD. NFS had been proved to show higher predictive performance in NAFLD cohort (20, 25, 26) and in this study, we used NFS to define AHF. NFS score >0.676 in the presence of NAFLD was assumed to have AHF. Formulas of FLI and NFS are as follows (21, 24, 25):


$$F\,L\,I=\frac{\left(e^{0.953\times l\,o\,g\,e\,\left(T\,G\right)+0.139\times B\,M\,I+0.781\times l\,o\,g\,e\,\left(G\,G\,T\right)+0.053\times W\,C-15.745}\right)}{1+e^{0.953\times l\,o\,g\,e\,\left(T\,G\right)+0.139\times B\,M\,I+0.718\times l\,o\,g\,e\,\left(G\,G\,T\right)+0.053\times W\,C-15.745}}\times 100;$$

NFS = $-1.675+0.037\times age+0.094\times BMI+1.13\times impairedfastingglycemiaordiabetes\left(yes=1,no=0\right)+0.99\times \frac{A\,S\,T}{A\,L\,T}ratio-0.013\times platelet-0.66\times albumin.$

Here, diabetes weas defined as glycated hemoglobin ≥6.5%, or self- reported diagnosed diabetes, or current use of antidiabetic medication (27).

### Covariates

Several potential confounding variables were selected as covariates based on the literature (28–31). We extracted these covariates, including age, poverty income ratio, waist circumference, alanine aminotransferase (ALT), aspartate aminotransferase (AST) and γ-glutamyl transferase (GGT), triglyceride (TG), total cholesterol (TC), High Density Lipoprotein cholesterol (HDL), body mass index (BMI), were measured as continuous variables; sex, race, marital status, education level, drinking status, smoking status, were assessed as categorical variables. A five-category system was used to divide race, namely Non-Hispanic Black, Non-Hispanic White, Mexican American, Other Hispanic, and Other Racial. The marital status of the participants was classified as never married, married/cohabitant, separated/divorced, and widowed. Level of education was categorized into “less than high school,” “high school,” “some college” and “college graduate or above.” The drinking status (32) was categorized as never (never drank 12 or more drinks in lifetime), former (drank 12 or more drinks in 1 year and didn’t drink last year, or didn’t drink last year but drank 12 or more drinks in lifetime), current light/moderate drinker (drank 1 or less drink per day for women or drank 2 or less drink per day for men on average over the past year), or current heavier drinker (drank more than 1 drink per day for women or more than 2 drinks per day for men on average over the past year). Smoking status (33) was classified into three categories: never (smoked less than 100 cigarettes in lifetime), former (smoked more than 100 cigarettes in lifetime and smoke not at all now), now (smoked moth than 100 cigarettes in lifetime and smoke some days or every day).

### Statistics

In the present study, all analyses accounted for the complex survey design of the NHANES and weighting variables. MEC subsample weight (WTMEC2YR for 2005–2016) was used, due to DII was used as major indicators in this study. Chi-squared test was used for comparing categorical variables and presented as numbers (n) and percentage (%). As for continuous variables, weighted *t*-test was used for comparing mean ± standard. Logistic regression was applied to determine associations between the DII, NAFLD, and AHF. A multivariable logistic regression model which included three models with increasing degrees of adjustment was used to examine the relationship between DII and NAFLD and AHF risk. Model I was the crude model. Model II was minimally adjusted for only race, sex, poverty income ratio, marital status, and education level. Model III was fully adjusted for race, sex, poverty income ratio, marital status, and education level, smoke status, BMI, waist circumference, AST, ALT, and GGT. A restricted cubic spline (RCS) was used to model the non-linear relationship between DII and NAFLD, and we used RCS with three knots at the 10th, 50th, 90th centiles to flexibly model the association of DII with NAFLD, however, DII is linearly related to AHF, and we did not execute RCS. Two-tailed *p*-values <0.05 were considered statistically significant. Statistical analysis software R version 4.1.2. was used for statistical analyses.

## Result

**FIGURE 1:**
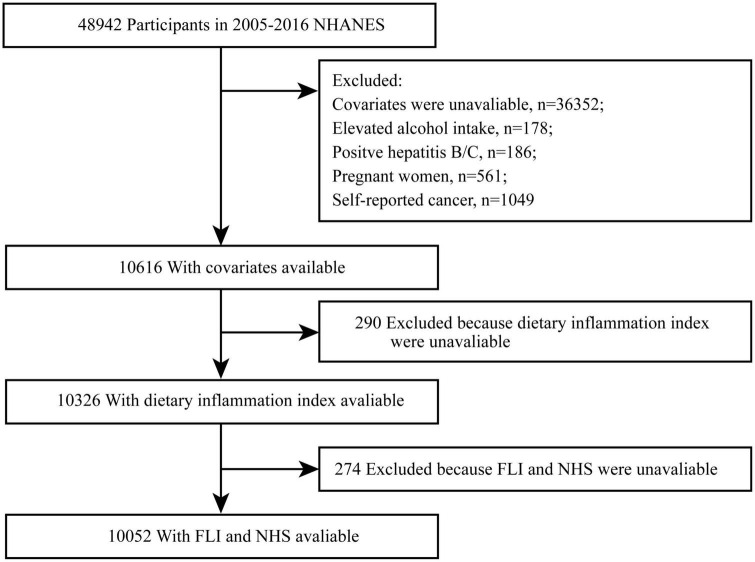
Participants flow chart.

A total of 10,052 subjects were invited to participant in the NHANES. These participants represented a weighted population of 72,033,969 non-institutionalized US. Figure 1 shows flowchart of the study.

Table 1 lists the participants characteristics according to DII quartiles, with scores ranging from -4.63 (most anti-inflammatory) to +5.47 (most pro-inflammatory). More than 65% of the population was non-Hispanic White, and rate decreased with ascending DII quartiles. Compared with quartile 1, higher DII group were associated with higher levels of female, separated/divorced, lower education level, heavy alcohol use, current smoke status, BMI, poverty income ratio, and waist circumference. DII also showed a significantly positive correlation with ALT, AST. Without adjustment for confounders, DII was positively associated with the risk of NAFLD and AHF.

**TABLE 1:** Characteristics of participants in the 2005–2016 continuous National Health and Nutrition Examination Survey (NHANES).

	Quartile 1	Quartile 2	Quartile 3	Quartile 4	*P*-value
Age (year)	46.03 (0.55)	46.55 (0.38)	45.66 (0.44)	46.32 (0.48)	0.31
**Sex (*n*, %)**					
Female	934 (37.59)	1,126 (45.59)	1,336 (54.22)	1,586 (65.67)	<0.0001
Male	1,579 (62.41)	1,385 (54.41)	1,178 (45.78)	928 (34.33)	
**Race (*n*, %)**					
Non-Hispanic White	1,179 (72.94)	1,121 (69.41)	1,092 (67.48)	1,092 (67.41)	<0.0001
Non-Hispanic Black	382 (6.95)	449 (9.14)	552 (11.92)	612 (13.50)	
Mexican American	455 (8.98)	437 (8.61)	416 (8.69)	359 (7.41)	
Other Hispanic	214 (4.44)	248 (5.33)	253 (5.49)	268 (5.99)	
Other Racial	283 (6.69)	256 (7.50)	201 (6.42)	183 (5.70)	
**Marital status (*n*, %)**					
Never married	456 (17.69)	418 (16.37)	509 (19.76)	504 (19.57)	<0.0001
Married/cohabitant	1,665 (70.03)	1,607 (67.78)	1,457 (61.91)	1,373 (59.00)	
Separated/divorced	288 (9.31)	340 (11.74)	362 (13.11)	402 (14.72)	
Widowed	104 (2.97)	146 (4.11)	186 (5.22)	235 (6.71)	
**Education level (*n*, %)**					
Less than high school	456 (11.23)	561 (14.56)	660 (18.96)	755 (20.30)	<0.0001
High school	472 (17.39)	527 (20.23)	602 (23.95)	681 (29.54)	
Some college	701 (28.51)	778 (33.15)	758 (32.90)	700 (31.40)	
College	884 (42.87)	645 (32.06)	494 (24.19)	378 (18.76)	
**Drinking status (*n*, %)**					
Never	275 (8.24)	310 (10.39)	355 (11.77)	438 (14.07)	<0.0001
Former	354 (11.60)	403 (13.35)	478 (15.82)	567 (19.55)	
Mild	995 (43.26)	906 (39.28)	773 (32.87)	670 (27.27)	
Moderate	373 (17.17)	396 (18.07)	417 (18.56)	348 (16.85)	
Heavy	516 (19.73)	496 (18.91)	491 (20.98)	491 (22.26)	
**Smoke status (*n*, %)**					
Never	1 425 (56.16)	1,424 (57.97)	1,386 (54.02)	1,368 (52.96)	<0.0001
Ever	663 (28.09)	671 (25.87)	559 (23.04)	515 (20.98)	
Current	425 (15.75)	416 (16.16)	569 (22.94)	631 (26.06)	
BMI (kg/m^2^)	28.15 (0.18)	28.84 (0.19)	29.40 (0.16)	29.72 (0.17)	<0.0001
Poverty income ratio (PIR)	3.37 (0.06)	3.14 (0.04)	2.88 (0.05)	2.61 (0.06)	<0.0001
Waist circumference	97.89 (0.50)	99.14 (0.53)	99.74 (0.42)	99.99 (0.41)	0.003
ALT (U/L)	26.39 (0.37)	25.72 (0.36)	25.08 (0.38)	23.93 (0.36)	<0.001
AST (U/L)	25.87 (0.31)	25.13 (0.30)	24.67 (0.28)	24.78 (0.52)	0.03
GGT (U/L)	25.87 (0.71)	26.28 (0.57)	28.22 (0.95)	27.03 (0.67)	0.17
TG (mg/dL)	125.41 (2.49)	130.27 (3.02)	128.59 (2.51)	124.77 (2.17)	0.31
TC (mg/dL)	192.72 (1.05)	194.61 (1.06)	194.38 (1.17)	193.21 (1.27)	0.54
HDL (mg/dL)	54.46 (0.42)	53.76 (0.49)	53.63 (0.51)	53.47 (0.43)	0.32
FLI	47.28 (0.94)	50.47 (1.07)	52.39 (0.83)	53.24 (0.77)	<0.0001
NFS	–2.02 (0.05)	–1.95 (0.04)	–1.95 (0.04)	–1.85 (0.04)	0.08
**NAFLD (*n*, %)**					
No	1,535 (61.94)	1,427 (56.82)	1,365 (54.89)	1,345 (53.89)	<0.0001
Yes	978 (38.06)	1,084 (43.18)	1,149 (45.11)	1,169 (46.11)	
**AHF (*n*, %)**					
No	2,421 (97.15)	2,387 (95.96)	2,367 (95.43)	2,322 (93.73)	<0.001
Yes	92 (2.85)	124 (4.04)	147 (4.57)	192 (6.27)	

For continuous variables, *p*-value was calculated by weighted *t*-test. For categorical variables, *p*-value was calculated by weighted chi-square test. Dietary inflammatory index (DII) quantile ranges: Quantile 1 = –4.63 to 0.27; Quantile 2 = 0.27–1.80; Quantile 3 = 1.80–3.03; Quantile 4 = 3.03–5.47.

Table 2 shows the association between DII and risk of NAFLD and AHF by a multivariable logistic regression model. Our results revealed that higher DII was associated with increased risk of NAFLD (model I, OR 1.08, 1.05–1.11 CI; model II, OR 1.09, 1.06–1.13 CI). After full adjustment (model III), DII was positively associated with the presence of NAFLD (OR 1.09, 1.06–1.13 CI), and this association meet the level of statistical significance. The association remained statistically significant after DII was grouped as quartiles. Participants in DII quartile 4 had a significantly 52% higher risk of NAFLD than those in DII quartile 1 (model III, OR 1.52, 1.27–1.83 CI, *p* trend <0.0001). After adjustment for potential confounding factors (model III), DII exhibited a significant positive association with AHF risk (OR 1.15, 1.07–1.23 CI, *p* trend <0.001).

**TABLE 2:** Association between dietary inflammatory index (DII) and the presence of non-alcoholic fatty liver disease (NAFLD) and advanced hepatic fibrosis (AHF) in the 2005–2016 continuous National Health and Nutrition Examination Survey (NHANES).

Dietary inflammatory index group	NAFLD	AHF
**Crude model (model I)**		
Continuous	1.08 (1.05, 1.11)	1.17 (1.09, 1.26)
**Quartile**		
1	1	1
2	1.24 (1.06, 1.45)	1.43 (0.98, 2.09)
3	1.34 (1.14, 1.57)	1.63 (1.09, 2.43)
4	1.39 (1.20, 1.62)	2.28 (1.57, 3.31)
*P* for trend	<0.0001	<0.0001
Minimally adjusted model (model II)*		
Continuous	1.09 (1.06, 1.13)	1.15 (1.07, 1.23)
**Quartile**		
1	1	1
2	1.24 (1.05, 1.46)	1.36 (0.92, 2.01)
3	1.37 (1.17, 1.60)	1.51 (1.01, 2.26)
4	1.48 (1.27, 1.74)	2.07 (1.42, 3.01)
*P* for trend	<0.0001	<0.001
**Fully adjusted model (model III)^†^**		
Continuous	1.09 (1.06, 1.13)	1.15 (1.07, 1.23)
**Quartile**		
1	1	1
2	1.26 (1.06, 1.49)	1.34 (0.90, 1.99)
3	1.35 (1.14, 1.61)	1.51 (1.00, 2.26)
4	1.52 (1.27, 1.83)	2.08 (1.44, 3.00)
*P* for trend	<0.0001	<0.001

Dietary inflammatory index (DII) quantile ranges: Quantile 1 = –4.63 to 0.27; Quantile 2 = 0.27–1.80; Quantile 3 = 1.80–3.03; Quantile 4 = 3.03–5.47.

*Adjusted for race, sex, poverty income ratio, marital status, education level.

^†^Adjusted for race, sex, poverty income ratio, marital status, education level, smoke status, BMI, waist circumference, AST, ALT, GGT.

Table 3 shows the association between DII and risk of NAFLD and AHF stratified by gender. DII levels were significantly and positively associated with the odds of NAFLD in males (Q4 vs. Q1: OR 1.62, 1.24–2.13 CI, *p* trend <0.001) and in females (Q4 vs. Q1: OR 1.37, 1.04–1.79 CI, *p* trend = 0.022) after full adjustment in model III. DII levels were associated with higher odds of AHF only in males (Q4 vs. Q1: OR 2.68, 1.63–4.41 CI, *p* trend <0.0001), and AHF risk was not significantly increased among Q2–Q4 participants compared with Q1.

**TABLE 3:** Survey weighted odds ratio (95% CI) for association between dietary inflammatory index (DII) and the presence of non-alcoholic fatty liver disease (NAFLD) and advanced hepatic fibrosis (AHF) stratified by sex in the 2005–2016 continuous National Health and Nutrition Examination Survey (NHANES).

Quartile for dietary inflammation index					
	Quartile 1	Quartile 2	Quartile 3	Quartile 4	*P*-trend
**NAFLD**					
**Males**					
Model I	1.00 (Ref.)	1.33 (1.07, 1.65)	1.52 (1.22, 1.90)	1.67 (1.33, 2.10)	<0.0001
Model II	1.00 (Ref.)	1.32 (1.05, 1.65)	1.56 (1.23, 1.98)	1.74 (1.36, 2.24)	<0.0001
Model III	1.00 (Ref.)	1.31 (1.03, 1.66)	1.48 (1.16, 1.91)	1.62 (1.24, 2.13)	<0.001
**Females**					
Model I	1.00 (Ref.)	1.25 (0.99, 1.57)	1.42 (1.14, 1.75)	1.59 (1.28, 1.99)	<0.0001
Model II	1.00 (Ref.)	1.13 (0.86, 1.48)	1.16 (0.91, 1.47)	1.21 (0.94, 1.54)	0.131
Model III	1.00 (Ref.)	1.18 (0.89, 1.55)	1.21 (0.93, 1.57)	1.37 (1.04, 1.79)	0.022
**AHF**					
**Males**					
Model I	1.00 (Ref.)	1.41 (0.87, 2.28)	1.79 (1.10, 2.93)	2.59 (1.58, 4.25)	<0.0001
Model II	1.00 (Ref.)	1.34 (0.83, 2.17)	1.76 (1.06, 2.92)	2.49 (1.49, 4.17)	0.001
Model III	1.00 (Ref.)	1.34 (0.81, 2.21)	1.75 (1.09, 2.82)	2.68 (1.63, 4.41)	<0.0001
**Females**					
Model I	1.00 (Ref.)	1.51 (0.85, 2.68)	1.56 (0.83, 2.92)	2.25 (1.29, 3.94)	0.004
Model II	1.00 (Ref.)	1.32 (0.71, 2.43)	1.25 (0.68, 2.32)	1.68 (0.96, 2.93)	0.061
Model III	1.00 (Ref.)	1.35 (0.73, 2.49)	1.21 (0.63, 2.33)	1.60 (0.90, 2.82)	0.123

Model I: Unadjusted. Model II: Adjusted for race, poverty income ratio, marital status, education level. Model III: Adjusted for race, poverty income ratio, marital status, education level, smoke status, BMI, waist circumference, AST, ALT, GGT.

**FIGURE 2:**
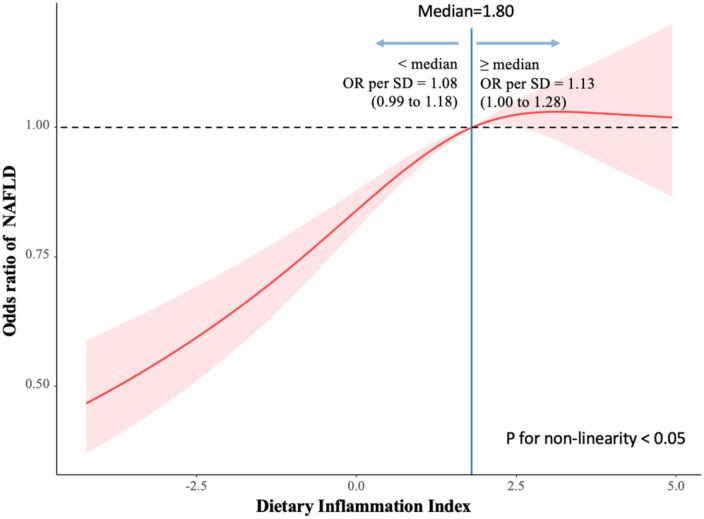
Association of dietary inflammatory index (DII) and non-alcoholic fatty liver disease (NAFLD) among adults. Reference point is median for NAFLD, with knots placed at 10th, 50th, 90th centiles of NAFLD distribution.

Figure 2 shows the association of DII and NAFLD among adults. we used restricted cubic spline to flexibly model and visualize the relation of DII and NAFLD among adults. The relationship started increase rapidly until around 1.80 and then started relatively flat afterward.

## Discussion

The present study found a pro-inflammatory diet as measured by the DII was associated with a deteriorating NAFLD and AHF risk profile in a nationally representative sample of US adults. In this cohort, the highest quartile of DII, the most pro-inflammatory diet, was positively associated with increased risk of NAFLD and AHF compared to the lowest quartile, the most anti-inflammatory diet. Additionally, in the subgroup analysis by sex basing on multiple logistic regression, DII exhibited a significant positive association with NAFLD, and AHF was not significantly in females.

Limited studies have investigated the link between the DII and NAFLD and AHF. Mazidi et al. (34) showed the significant association between the inflammatory potential of diet and FLI by resulting that individuals with the highest DII quartile had nearly a 6-fold higher likelihood of fatty liver (OR 5.97, 4.44–8.02 CI) compared with those with the lowest DII quartile. NFS is commonly used for detecting fibrosis among NAFLD patients and has been shown to be as accurate as a liver biopsy in stratifying patients at risk for liver-related morbidity and mortality (35). We used NFS to define AHF in this study. To date, the association between AHF and DII has not been previously investigated. We found that AHF were positively associated with DII scores. Highest DII quartile had higher odds than those in the lowest DII quartile after full adjustment (OR 2.08, 1.44–3.00 CI, *p* trend <0.001, Table 2). Then, subgroup analysis was performed for gender, DII was significantly correlated with higher risk of NAFLD in both genders, and with higher risk of AHF in males but not in females. Further studies in independent populations would be essential to support these findings.

Our results highlight that anti-inflammatory diets were associated with lower risks of NAFLD and the prevention of its progression to advanced fibrosis. Kenđel et al. (36) investigated the relation between energy-reduced anti-inflammatory diet and liver status using a two-arm randomized controlled trial, founding that FLI was 14.3% lower after 6 months energy-restricted anti-inflammatory diet and for FIB-4 (a validated diagnostic index, to estimating the liver fibrosis possibility) was 2.5% lower. Another study included (37) 8,520 adults in western Iran and found that more pro-inflammatory diet in participants was associated with higher FLI. Similarly, Li and Chen (38) suggested that anti-inflammatory diets might have hepatoprotective effects that reduce the risk of NAFLD.

The major highlights of this study are the relatively large and well-designed population-based sample, the results can be extrapolated to the entire population. Due to the strong standardization of the NHANES study procedures, the measurement and information biases are very low (39). Our findings suggest that a more specific focus on the DII level for NAFLD patients is required, and AHF patients, especially in males.

There were several limitations to this study as follows. First, the cross-sectional nature of NHANES severely constrains causal inferences. Second, in the absence of gold standard techniques to diagnose NAFLD and AHF, we used non-invasive diagnosis indexes (i.e., the FLI and NFS). Third, the lack of data on physical activity makes it impossible to predict whether it would be a potential predictor. However, combined with previous study (34), this predictor may not have a decisive effect on outcomes. Finally, a 24 h dietary recall is used to calculate DII, limiting the ability to accurately describe individuals’ habitual diets, and recall bias is inevitable. In addition, the non-availability of 18 food parameters for calculating the DII score may be a limitation of the study as well. Nevertheless, it has been shown in previous studies that the absence of these missing components is unlikely to have a major impact on DII scores since they are consumed infrequently in the US population (40). Thus, further studies needed to clarify the causal relationship and to confirm these findings.

In conclusion, data from this study suggesting a possible association between NAFLD and AHF risk with the DII. We suggest the anti-inflammatory diet can be a step of NAFLD and AHF management and be considered to prevent NAFLD and AHF in adults. However, further longitudinal studies, larger sample sizes and repeated measures are necessary to validate and verify the current evidence.

## Data availability statement

We analyzed publicly available datasets in this study. The datasets presented in this study can be found in online repositories. The names of the repositories can be found below: Centers for Disease Control and Prevention (CDC) National Health and Nutrition Examination Survey (NHANES), https://wwwn.cdc.gov/nchs/nhanes/Default.aspx, 2005–2016. Using deidentified data to conduct the secondary analysis was officially classified as exempt by the Albert Einstein College of Medicine Institutional Review Board.

## Ethics statement

Ethical review and approval was not required for the study on human participants in accordance with the local legislation and institutional requirements. The patients/participants provided their written informed consent to participate in this study.

## Author contributions

ZhL and JL were responsible for the conception and design of the study. ZZ, LW, and ZiL wrote the first draft of the manuscript, interpreted the data, and wrote the final version. WeY, JC, XZ, WaY, and JL performed the statistical analysis and interpreted the analysis. ZhL, JL, JC, XZ, and WeY were critically revised the manuscript. ZhL obtained public funding. All authors critically revised the article for important intellectual content and approved the final version.
